# Monitoring the efficacy of mutated *Allium sativum* leaf lectin in transgenic rice against *Rhizoctonia solani*

**DOI:** 10.1186/s12896-016-0246-0

**Published:** 2016-03-01

**Authors:** Prithwi Ghosh, Senjuti Sen, Joydeep Chakraborty, Sampa Das

**Affiliations:** Division of Plant Biology, Bose Institute, Centenary Campus, P1/12, CIT Scheme, VIIM, Kankurgachi, Kolkata, 700054 West Bengal India

**Keywords:** Antifungal protein, Mutant *Allium sativum* Leaf Agglutinin (mASAL), *Rhizoctonia solani*, Sheath blight, Transgenic rice

## Abstract

**Background:**

Rice sheath blight, caused by *Rhizoctonia solani* is one of the most devastating diseases of rice. It is associated with significant reduction in rice productivity worldwide. A mutant variant of mannose binding *Allium sativum* leaf agglutinin (mASAL) was previously reported to exhibit strong antifungal activity against *R. solani*. In this study, the *mASAL* gene has been evaluated for its *in planta* antifungal activity in rice plants.

**Results:**

mASAL was cloned into pCAMBIA1301 binary vector under the control of CaMV35S promoter. It was expressed in an elite indica rice cv. IR64 by employing *Agrobacterium tumefaciens*-mediated transformation. Molecular analyses of transgenic plants confirmed the presence and stable integration of *mASAL* gene. Immunohistofluorescence analysis of various tissue sections of plant parts clearly indicated the constitutive expression of mASAL. The segregation pattern of *mASAL* transgene was observed in T_1_ progenies in a 3:1 Mendelian ratio. The expression of mASAL was confirmed in T_0_ and T_1_ plants through western blot analysis followed by ELISA. *In planta* bioassay of transgenic lines against *R. solani* exhibited an average of 55 % reduction in sheath blight percentage disease index (PDI).

**Conclusions:**

The present study opens up the possibility of engineering rice plants with the antifungal gene *mASAL*, conferring resistance to sheath blight.

## Background

Rice (*Oryza sativa* L.) is a major food crop for more than half of the global population, although it experiences various biotic and abiotic stresses throughout its life cycle. Sheath blight is considered to be an important disease of rice next to the blast disease. It is caused by a cosmopolitan, soil-borne basidiomycete necrotrophic fungus *Rhizoctonia solani* Kühn (teleomorph: *Thanatephorus cucumeris*). Sheath blight results in severe damage and reduces the rice yield by 8–50 % in the rice-growing countries of Asia [1]. In India, it causes about 20 % loss of rice yield [2]. Moreover, the damage caused by sheath blight has become more alarming nowadays, due to the increased use of semi-dwarf, nitrogen-responsive and high-yielding varieties [3]. It is difficult to manage sheath blight because of the wide host range of this pathogen, high pathogenic diversity and its ability to survive in soil for a long time [4]. In addition, attempts to control sheath blight through conventional breeding are not possible as there is no such records of genetic resistance to sheath blight among cultivars and wild races of rice [5]. Application of fungicide is a common practice to control plant diseases. Control through chemical methods significantly increases production cost and poses serious health and environmental threats. In addition, the emergence of fungicide-resistant pathogens demands effective antifungal candidate genes.

In view of the above, the introduction of antifungal genes into rice cultivars may be the suitable method for the fight against sheath blight. Till date, an array of antifungal proteins has been biotechnologically exploited to generate transgenic plants conferring resistance to sheath blight, including chitinases [6–8] thaumatin-like proteins [9] nonspecific lipid transfer proteins [10] and plant defensin [11].

Lectins are carbohydrate-binding, heterologous group of proteins that bind reversibly to specific mono-or oligosaccharides, possessing at least one non-catalytic domain [12]. In plant-pathogen interactions, plant lectins provide plants with a passive defense system against various pathogens by their ability to bind to specific carbohydrates [13]. Several plant lectins have been exploited to develop insect resistant plants [14–16] and few lectins are reported to manifest antifungal activity [17–21]. *Allium sativum* Leaf Agglutinin (ASAL) is a mannose-binding 25-kDa homodimeric lectin, isolated from garlic (*Allium sativum* L.) leaves and showed potent insecticidal activity against homopteran pests [22–24]. A stable monomeric mutant variant of *Allium sativum* Leaf Agglutinin (mASAL) was generated by radically changing the oligomerization level of ASAL by the insertion and replacement of five amino acid residues (−DNSNN-). Interestingly, this 12-kDa mutant ASAL exhibits an *in vitro* antifungal activity against a broad spectrum of plant pathogenic fungi including *R. solani* [25].

The exact mode of action of mASAL on *R. solani* is not clear, however, its antifungal activity was found to be associated with the alteration of cell membrane permeability of the fungus [25]. In addition, a ligand blot assay of total protein from *R. solani* with mASAL detected the presence of several interactors. Hence, binding of mASAL with the interactors is assumed to have an adverse influence on the different key metabolic pathways of *R. solani* [26].

Nevertheless, there is a growing concern among the scientific community as well as among consumers regarding the risk of allergenicity induced by any foreign or engineered protein expressed in genetically modified plants. Thus, while targeting any new genes in crop plants, the possibility of allergenicity and toxicity associated with the gene product must be considered. So, both *in vitro* and *in vivo* safety assessment of mASAL was performed following the FAO/WHO guidelines (2001) [27]. The results revealed that mASAL appears to be safe and poses no unfavourable features towards model animals and humans in terms of toxicity and allergenicity [28]. In view of the strong antifungal activity as well as biosafety, mASAL stands out to be a promising candidate for engineering agronomically important crop plants. In this study, we report the stable transformation of an elite indica rice, IR-64, with the antifungal gene *mASAL*. Our results showed that *in planta* expression of mASAL significantly improved resistance to sheath blight in comparison to wild-type rice plants.

## Results

### Development of *mASAL* expressing transgenic plants

**Fig. 1:**
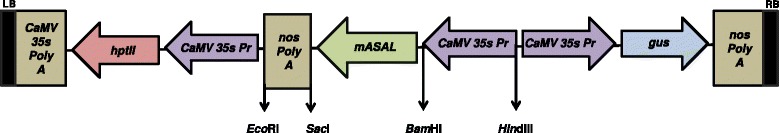
Schematic representation of the T-DNA segment of plant expression vector. CaMV35SPr., cauliflower mosaic virus 35S promoter; CaMV35S polyA, cauliflower mosaic virus 35S terminator; *mASAL*, mutant *Allium sativum* leaf agglutinin; *hptII*, hygromycin phosphotransferase II; *gus,* β-Glucuronidase; nos polyA, nopaline synthase terminator; LB, left border of T-DNA; RB, right border of T-DNA

**Fig. 2:**
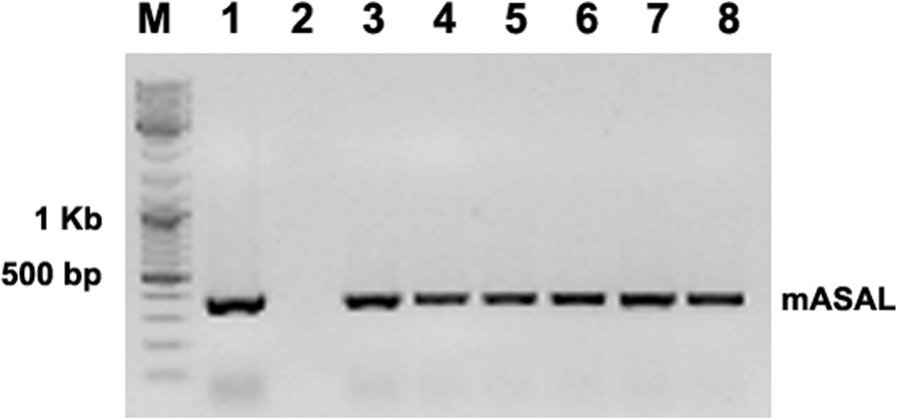
PCR analysis for the *mASAL* gene in randomly chosen T_0_ progenies. Lane 1 showing amplification of *mASAL* gene at ~333 bp as positive control (pCAMBIA1301*mASAL* plasmid); Lane 2 represents negative control (untransformed IR64); Lane 3–8 represents mASAL transgenic plants of lines RSR4, RSR7, RSR20, RSR28, RSR34 and RSR45, respectively; Lane M, DNA ladder as molecular weight marker

The plant expression cassette comprising cauliflower mosaic virus 35S (CaMV35S) promoter, a 333 bp *mASAL* coding sequence and a nos terminator was cloned into *Hin*dIII/*Eco*RI site of pCAMBIA1301. The recombinant clone was designated pCAMBIA1301*mASAL* (Fig. 1) and used in plant transformation for the constitutive expression of mASAL. The resulting plasmid was introduced into *Agrobacterium tumefaciens* (LBA4404) for the genetic transformation experiments. The indica rice cv. IR64 has been used in the present study for developing transgenic rice plants. Preliminary screening of the randomly selected ten T_0_ transformants, regenerated from hygromycin resistant calli, was performed by PCR amplification of the *mASAL* gene. PCR analysis from the untransformed plants (control) showed no amplification while an amplified fragment of ~333 bp was detected in transformed leaf samples (Fig. 2). A total of six independently transformed hygromycin resistant, PCR positive T_0_ plants (RSR4, RSR7, RSR20, RSR28, RSR34 and RSR45) were grown and multiplied to T_1_ plants for further analyses. All of these transformed plants were morphologically similar to the non-transformed controls with respect to the vegetative growth, flowering and seed setting.

### Stable integration and inheritance of *mASAL* gene

**Fig. 3:**
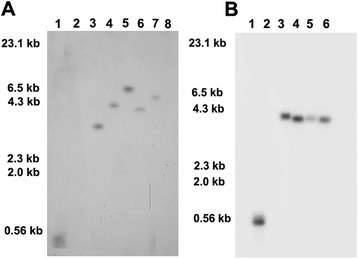
Southern blot analysis of the PCR positive T_1_ and T_2_ transformants. **a** Southern blot analysis of *Hin*dIII digested genomic DNA from leaves of six individual T_1_ progeny plants of corresponding T_0_ lines [RSR4 (T_1_3), RSR7 (T_1_4), RSR20 (T_1_2), RSR28 (T_1_1), RSR34 (T_1_8) and RSR45(T_1_6)] in lanes 3–8, respectively; lane 8, RSR45(T_1_6), a segregating progeny showing absence of *mASAL* gene cassette. **b** Southern blot analysis of *Hin*d*III* digested genomic DNA from leaves of four T_2_ progenies of RSR7 (T_1_4) plant in lanes 3–6, respectively. *mASAL* gene was used as positive control (lane 1) and *Hin*dIII digested genomic DNA from control plants as negative control (lane 2). Approximate DNA molecular weight markers are indicated on the left

After selfing, seeds were collected from six independent T_0_ plants. The integration of the transgene, in PCR positive T_1_ lines, were confirmed by Southern blot hybridization. Genomic DNA was extracted from T_1_ progenies of respective T_0_ plants and digested with *Hin*dIII, as there is only a single *Hin*dIII site at the 5’ end of the *mASAL* gene cassette. After hybridization using radiolabelled *mASAL* specific gene probe, all the lines documented different banding patterns, suggesting the independent integration event in each line. Plant number RSR4 (T_1_3), RSR7 (T_1_4), RSR20 (T_1_2), RSR28 (T_1_1) and RSR34 (T_1_8) showed a single copy of transgene insertion and one plant [RSR45 (T_1_6)] did not show any integration (Fig. 3a). RSR7 (T_1_4) was further analyzed in the next generation. Four T_2_ progenies of RSR7 (T_1_4) demonstrated that the integration patterns were same as the parental line (Fig. 3b). Further analyses were carried out with plants having single copy insertions. PCR screening for *mASAL* gene using DNA isolated from randomly chosen T_1_ seedlings demonstrated that segregation of the *mASAL* gene followed 3:1 Mendelian segregation pattern and the observed ratio was validated using *χ*^2^ test (Table 1).

**Table 1:** Segregation analyses of T_1_ plants derived from selfed T_0_ plants

T_0_ plant no	Number of T_1_ seeds tested	*mASAL* ^PCR+^	*mASAL* ^PCR-^	Observed ratio	*χ* ^2^ -value	*P* - value
RSR 4	27	19	8	2.37:1	0.308	0.5789
RSR7	32	25	7	3.5:1	0.166	0.6837
RSR20	20	14	6	2.3:1	0.266	0.606
RSR28	22	17	5	3.4:1	0.06	0.0865
RSR34	38	28	10	2.8:1	0.034	0.8537
RSR45	33	26	7	3.7:1	0.252	0.6157

### Expression of mASAL in transgenic rice

**Fig. 4:**
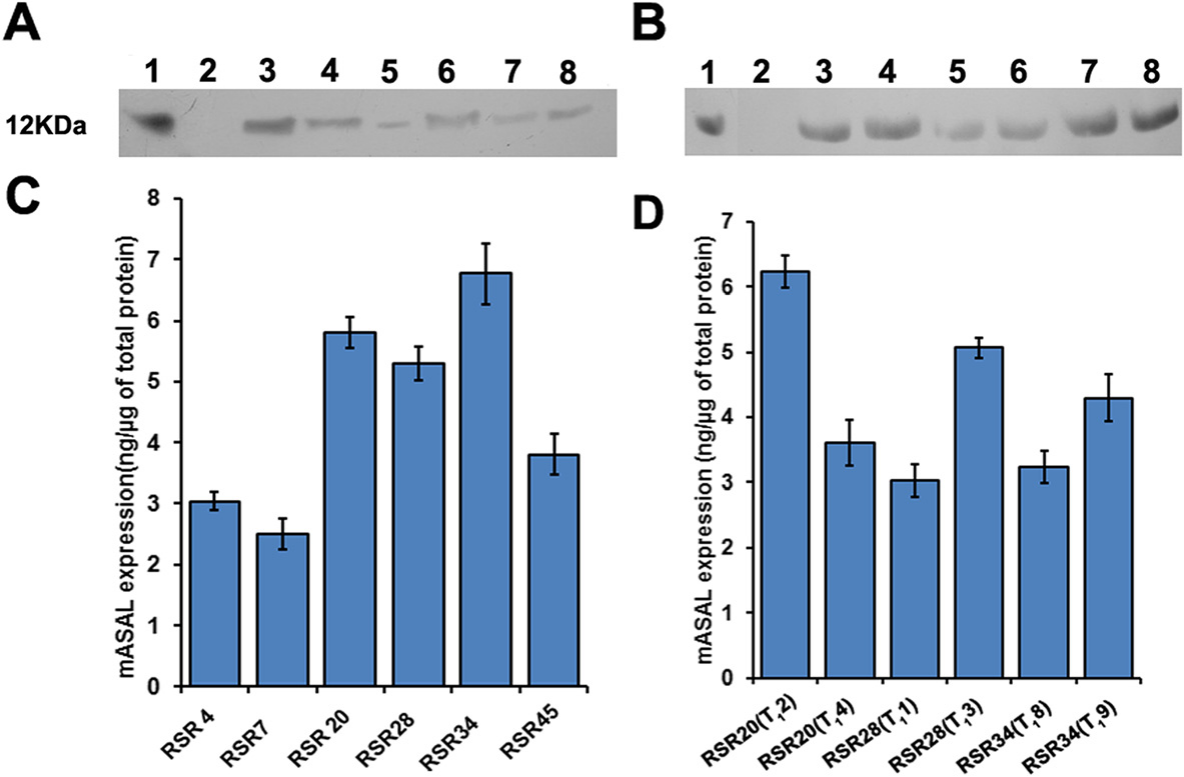
Detection of mASAL in transgenic rice. **a** Western blot analysis of total protein extracts isolated from the leaves of 35S*mASAL,* six independent T_0_ transgenic rice lines (RSR4, RSR7, RSR20, RSR28, RSR34, and RSR45 in lane 3–8) and **b** six T_1_ progeny plants of RSR20 (T_1_2, T_1_4), RSR28 (T_1_1, T_1_3) and RSR34 (T_1_8,T_1_9) (lane 1–8). Lane 1, purified mASAL; lane 2, protein extract from untransformed control plant. **c** ELISA analysis for expression of mASAL in total soluble protein in six T_0_ transformants (RSR4, RSR7, RSR20, RSR28, RSR34 and RSR45) and **d** in six T_1_ progeny plants of line RSR20 (T_1_2, T_1_4), RSR28 (T_1_1, T_1_3), and RSR34 (T_1_8, T_1_9). The bars represent the mean ELISA reading of three replicas per sample of three experiments

Western blot analysis clearly indicated the presence of ~12-kDa band of expressed mASAL protein separated in 15 % sodium dodecyl sulphate polyacrylamide gel electrophoresis (SDS-PAGE) when probed with the anti-mASAL polyclonal antibody. No such band was observed in the untransformed control plants. All of the six T_1_ progeny plants and their corresponding T_0_ parental lines were able to express the ~12-kDa mASAL protein (Fig. 4a, b). The amount of mASAL in leaves of T_0_ and T_1_ rice plants was quantified using indirect ELISA (Fig. 4c, d). Expression levels ranged between 0.25 and 0.67 % of total soluble protein, in the leaf extracts of transgenic lines.

### Immunohistofluorescence localization of mASAL in transgenic plants

**Fig. 5:**
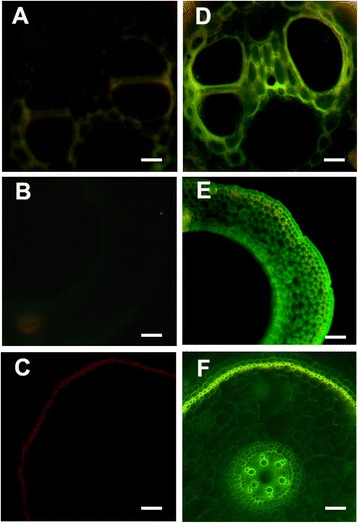
Immunohistoflourescence localization of mASAL in transgenic rice. Transverse sections were prepared from tissues of mASAL expressing transgenic lines and control untransformed plants. Panels **a**, **b** and **c** represent the transverse sections of leaf, stem and root of control plants. Panels **d**, **e** and **f** represent the transverse sections of leaf, stem and root of mASAL expressing transgenic lines. Tissue sections were treated with anti-mASAL anti-serum as primary antibody and FITC-conjugated anti-rabbit IgG as secondary antibody. The presence of mASAL is indicated by the green fluorescence. Bar represents 10 μm

Immunohistofluorescence analysis of expressed mASAL was studied by treating transverse sections of both untransformed and transformed rice stems, leaves and roots with the anti-mASAL primary antibody, followed by FITC-conjugated anti-rabbit IgG. Untransformed plants were used as negative control. Untransformed and transformed plant sections were processed in the same manner. Fluorescence microscopy demonstrated the constitutive expression of mASAL in all tissue types of stem, root and leaf sections of transgenic rice plants as depicted in Fig. 5d, e and f, respectively. Whereas, the untransformed plants showed no fluorescence after treatment with the antibodies (Fig. 5a, b and c).

### Assessment of disease tolerance of transgenic rice

**Fig. 6:**
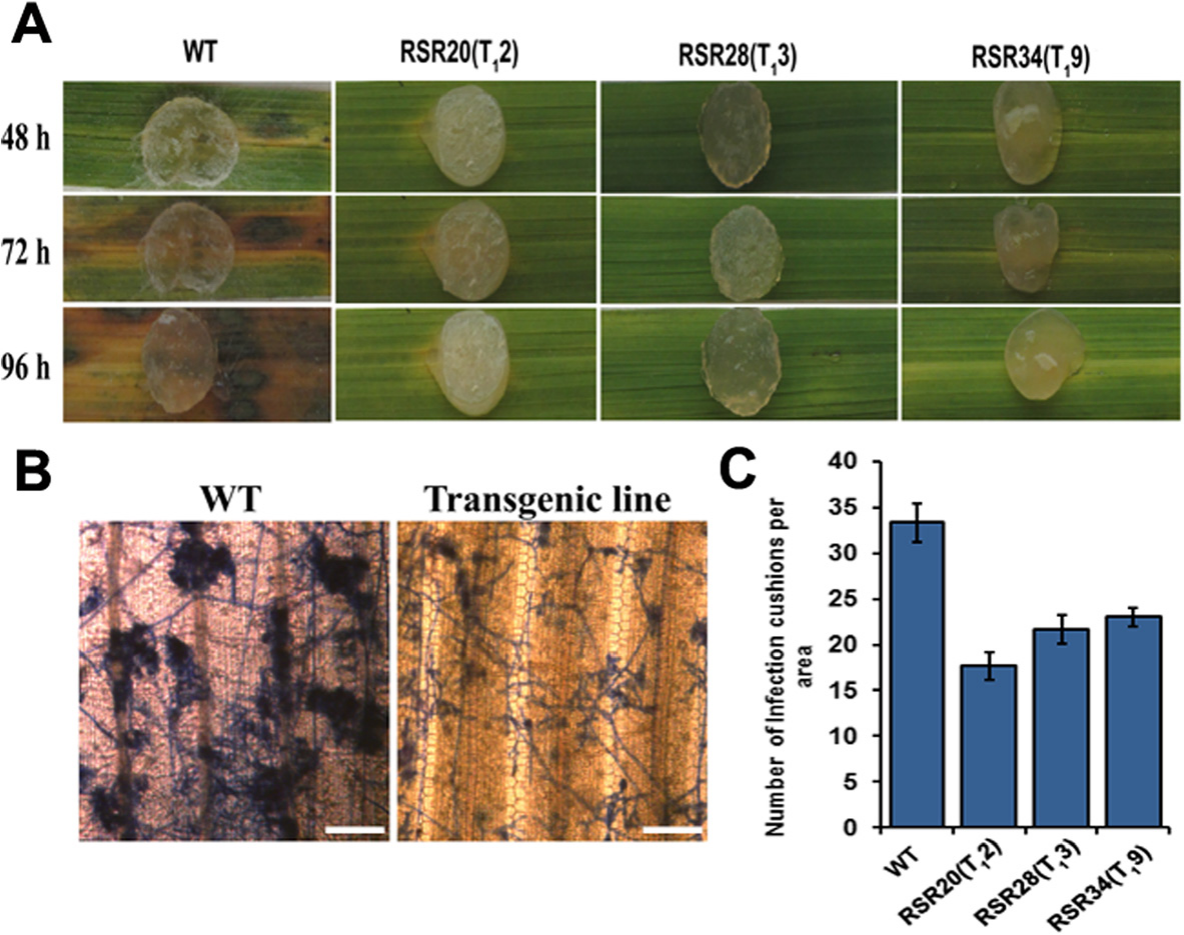
Assessment of sheath blight resistance by detached leaf bioassay. **a** Representative images of the lesion formation in leaves of non-transgenic control and transgenic lines [RSR20(T_1_2), RSR28(T_1_3) and RSR34(T_1_9)] following inoculation with *R. solani* at 48, 72 and 96 hai, respectively. **b** Photomicrograph of a portion of trypan blue stained control and transgenic leaf at 72 hai showing infection cushions. Magnification bar represents 20 μm. **c** Graphical representation of number of infection cushions in control and transgenic plants [RSR20(T_1_2), RSR28(T_1_3) and RSR34(T_1_9)] at 72 hai. Each data point represents the average of three individual measurements with standard deviations as error bars

To determine the functional relevance of mASAL expression in rice, transgenic rice plants were tested for resistance against a virulent strain of *R. solani* AG-1-1A. Detached leaf bioassay with *R. solani,* showed sheath blight symptoms appeared within 48 h after inoculation (hai) with yellowing of margins surrounding the area of inoculum in untransformed control leaves. The lesion was found to extend progressively at 72 and 96 hai. In contrast, leaves of transgenic plants almost remained green and fresh with respect to the non-transgenic control; where minimal yellowing of leaves was observed at 72 and 96 hai (Fig. 6a).

In addition, to substantiate the results of detached leaf bioassay, trypan blue staining and microscopic observations of *R. solani* hyphae were carried out at 72 hai using a light microscope. Extensive colonization of fungal hyphae, forming prominent infection cushions was observed in the leaves of control wild-type plants. In contrast, under the same experimental conditions, no such intensive fungal colonization was observed in the leaves of mASAL expressing plants at 72 hai (Fig. 6b). Transgenic plants were further evaluated by comparing the number of infection cushions in the leaves of transgenic and non-transgenic control plants. This indicated a prominent reduction in the number of infection cushion in the transgenic lines with respect to the non-transgenic control plants (Fig. 6c)*.*

**Fig. 7:**
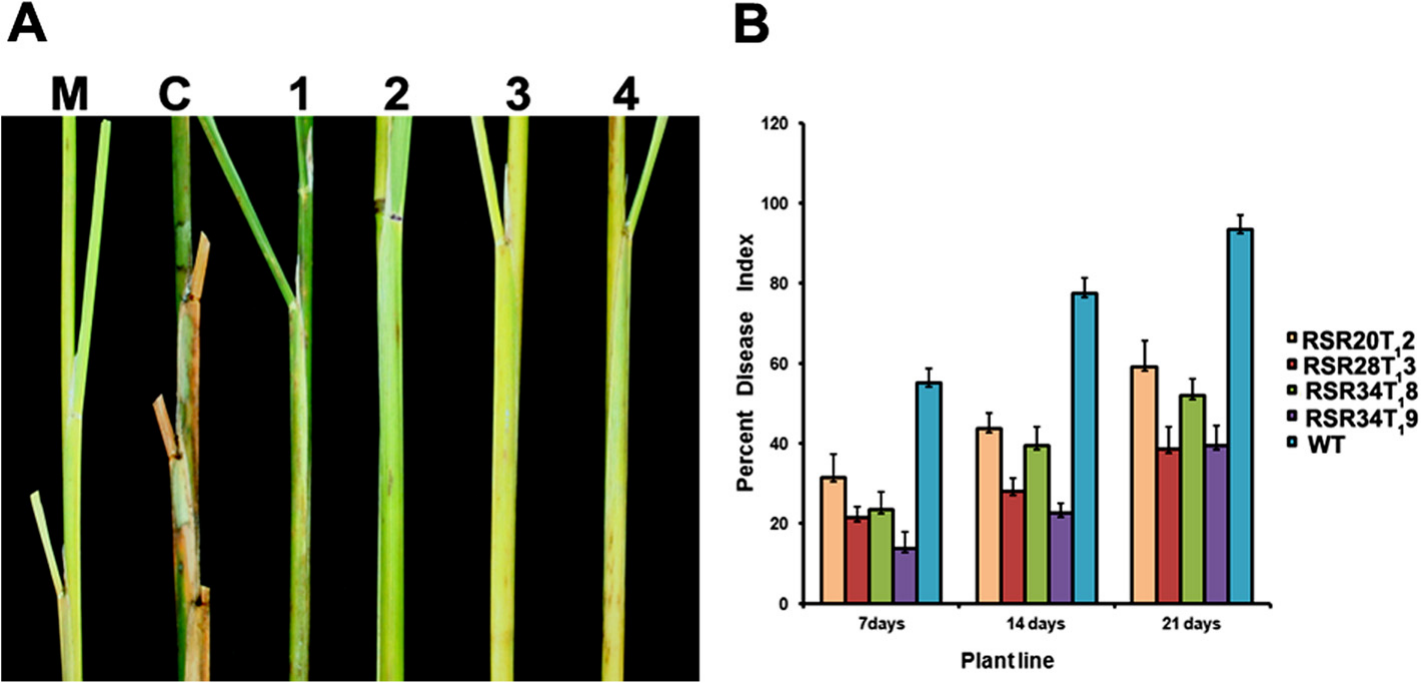
Resistance in transgenic rice plants constitutively expressing mASAL. **a** Representative images showing whole plant infection assay of control and transgenic plants at 7dpi. M, negative control without *R. solani* inoculam; C, untransformed plant infected with *R. solani*; 1,2,3 and 4 are infected transgenic plants of the lines RSR20(T_1_2), RSR28(T_1_3), and RSR34(T_1_8,T_1_9), respectively. Each plant represents the infected portion of one representative tiller from each transgenic line. **b** Response of transgenic lines and non-transgenic control to the sheath blight infection was scored as the relative lesion height and expressed as the percentage disease index (PDI) in transgenic line and wild-type plants. The values were shown as the mean ± standard error (SE). Individual tillers were considered as separate replications. Data sets were analyzed for significant difference using ANOVA (*P* < 0.05)

To further validate the results obtained by using the detached leaf bioassay, *in planta* inoculation experiments were carried out. The progression of sheath blight infection in the control plant in comparison to the transgenic lines at 7 days post-inoculation (dpi), is shown in Fig. 7a. The transgenic lines exhibited a delayed symptoms appearance and reduced disease intensity compared to that of the control plants. In the whole plant bioassay, the mASAL expressing transgenic lines recorded a lower percentage disease index (PDI) compared with the control plants. The PDI was scored after first, second and third weeks after *R. solani* infection in the control and the transgenic T_1_ plants [RSR20, RSR28 and RSR34] (Fig. 7b). The PDI in the control plants, which was 55.1 after week one, progressed to 77.4 and 93.4 after second and third weeks, respectively. The PDI of the T_1_ transgenic plants (RSR20, RSR28 and RSR34), which was in the range of 13.82–31.5 in the first week, changed from the range of 22.68–43.9 to 39.3–59.07 in the second and third weeks, respectively, after infection. Thus, the PDI of the transgenic lines were significantly lower than the control at all the three-time points (*P* < 0.05).

## Discussion

Engineering fungal resistance in plants is a promising strategy in terms of cost, efficacy and sole dependence on pesticides. In this study, mASAL was selected as it was found to exhibit strong antifungal activity against *R. solani* [25]. Furthermore, the safety evaluation also indicated that mASAL does not pose a risk of food allergy [28]. mASAL was engineered into rice through *Agrobacterium*-mediated transformation protocol to monitor its efficacy against sheath blight. GUS-positive, putatively transformants, regenerated from the hygromycin resistant calli were subjected to molecular analyses. PCR analysis using *mASAL* specific primers showed amplification at ~333 bp region in six transformants, confirming the integration of the gene. None of the transgenic lines showed any phenotypic alteration.

Multiple copies of a transgene(s) inserted in single or multiple loci often lead to the problems of co-suppression, silencing, sterility, non-Mendelian inheritance of transgene and instability over generations [29]. Thus, single copy integration of transgene(s) is always preferred to achieve predictable patterns of transgene inheritance and to overcome the problem of gene silencing in transgenic plants [30]. It was reported previously that the use of multicopy binary vectors may integrate multiple copies of T-DNA into the plant genome, which had a propensity for silencing to a greater extent than do the single integrated copies [31]. Therefore, the use of multicopy binary vectors introduced two common problems associated with plant transformation, multiple integrated transgene copy number and vector backbone integration. Nowadays, low-copy-number T-DNA binary vectors are being used to eliminate these problems [32]. The correlation between the transgene copy and the level of gene expression is known to be complex. Some reports showed that the transgene copy number is inversely correlated to the expression levels [33]. To simplify the transgenic analysis and to validate the true transgenic nature of the primary transformants, the inheritance of *mASAL* gene was analyzed by Southern blot hybridization in stringently selected T_1_ plants and their T_2_ progenies. Southern blot analysis with the *mASAL* probe, in *Hin*dIII digested genomic DNA of transformants revealed the stable integration of *mASAL* in different transgenic lines. Five out of six independent transgenic events representing the randomly selected T_1_ progeny plants of corresponding T_0_ lines showed single copy integration. One of the representative progeny plant i.e. T_1_ (T_1_6) of the corresponding RSR45 T_0_ line did not show the integration of *mASAL* gene cassette. This could be due to the segregation of the transgene in T_1_ generation. One of the lines [RSR7(T_1_4)] was further analysed for the inheritance of the *mASAL* in T_2_ generation through Southern blot hybridization, which showed that copy number of transgene integration among the T_2_ plants was identical to their parental line. This suggests that no rearrangement of the *mASAL* gene has occurred during the segregation. Furthermore, the Chi-square analysis was conducted for testing the segregation of transgene in T_1_ generation. This revealed that the observed ratio fits well to the expected 3:1 ratio.

The expression of mASAL in the transgenic lines T_0_ and T_1_ was analyzed by western blot analysis and indirect ELISA. Western blot analysis of the transgenic lines confirmed the constitutive and stable expression of mASAL. ELISA was carried out to monitor the quantitative expression of mASAL in T_0_ and T_1_ plants. The expression level ranged between 0.25 and 0.67 % of total soluble protein, in different transformants, which suggests that the transgene has integrated randomly at different transcriptionally active sites within the plant genome. The immunohistoflourescence localization revealed that mASAL was strongly expressed in various plant parts like stems, leaves and roots. However, the constitutive expression of transgenes may increase the metabolic load and the energy cost of the transgenic plants. Therefore, to avoid the unwanted expression of the target gene in non-target organs and tissues and to reduce the severity of sheath blight infection, it is highly desirable to express the target genes at the specific site of infection.

We further assessed the efficacy of mASAL on rice against sheath blight. Both the detached leaf and whole plant bioassay showed that mASAL-expressing transgenic rice exhibited significant resistance to sheath blight. The inhibitory effect of mASAL in infection cushions formation at 72 hai can be directly correlated with the expression level of mASAL in the three transgenic lines [RSR20 (T_1_2), RSR28 (T_1_3) and RSR34 (T_1_9)]. In addition, in the whole plant bioassay the two transgenic lines [RSR20 (T_1_2), and RSR28 (T_1_3)] exhibited the variation between the degree of sheath blight resistance and the expression levels of mASAL at 7, 14 and 21dpi. However, a direct correlation with the expression of mASAL was observed in the transgenic line [RSR34 (T_1_8, T_1_9)] at the above three time points.

The three mASAL expressing transgenic T_1_ lines [RSR20(T_1_2), RSR28(T_1_3) and RSR34(T_1_9)] exhibited enhanced resistance. Unlike the larger lesions of non-transgenic leaves, the transgenic leaves documented the formation of defensive yellowing at the site of inoculation. The infection cushion plays a crucial role in the disease progression by enzymatic degradation and physical penetration through the leaf surface [34]. Interestingly, strong support of resistance to sheath blight was evident from the growth suppression and reduction in the number of infection cushions observed in the leaf surface of transgenic lines. Earlier reports also showed suppression of fungal invasive hyphae in transgenic rice expressing antifungal proteins [35, 36]. Definitive proof of sheath blight resistance came from the whole plant bioassay, which was performed according to Park et al. [37]. Upon inoculation with *R. solani*, the mASAL expressing transgenic lines recorded a lower PDI as compared to that in the control. In transgenic plants, sheath blight symptoms development was delayed, and small brownish lesions started appearing on 7 dpi. The delayed occurrence and relatively slow enlargement of lesions coupled with extensive browning (a host defense reaction) around the lesions in transgenic plants suggest enhanced resistance against *R. solani.* On average, a 55 % reduction in the PDI in mASAL expressing plants relative to non-transgenic plants was observed. In the present study, the reduction in the average PDI was more or less comparable, or higher than the previous reports. A 25 % reduction in disease severity was observed in transgenic rice co-expressing ribosome-inactivating protein and a rice chitinase relative to the control plants [38]. In another report, a 45 % reduction in disease symptoms was observed in transgenic rice expressing *Rs-AFP2* defensin gene [11]. More or less 50 % reduction in the PDI was observed in *Osoxo4*-overexpressing plants [35].

## Conclusions

In summary, the present study highlights the efficacy of mASAL against *R. solani* by developing mASAL expressing transgenic rice plants. The selected transgenic lines displayed an improved resistance to sheath blight. Therefore, the use of this novel antifungal gene may appear as a promising strategy for future management of other fungal pathogens. Additional research is also needed to focus on the overall analysis of mASAL expressing plants in terms of agronomic traits and monitoring its efficacy against greater diversity of pathogens under natural field conditions.

## Methods

### Plant material

Rice (*Oryza sativa* L.) cv. IR64 seeds obtained from Regional Rice Research Station, Chinsurah, West Bengal, India were used for the plant transformation.

### Fungal material and culture conditions

*R. solani* (MTCC code-4633) culture obtained from IMTEC, Chandigarh, India was used in this study. The fungal pathogen was routinely maintained aseptically on potato dextrose agar (PDA) plates at 28 °C, by subculturing after 14 days in dark.

### Vector constructions and plant transformation

The 333 bp *mASAL* gene was amplified using the forward primer (F1): 5’AGCTGGATCCATGGCCAGCAACCTACTGACGAAC3’ and reverse primer (R1): 5’ AATGAGCTCCTAGGTACCAGTAGACCAAAT 3’ containing the *Bam*HI and *Sac*I sites respectively. The gene was cloned into corresponding restriction site of pCAMBIA1301 in between CaMV 35S promoter and nos terminator [39]. The binary vector, pCAMBIACaMV35S*mASAL*, was maintained in the DH5α strain of *E. coli* and mobilized to *A. tumefaciens* LBA4404. The binary vector comprised *gus*A reporter gene and selectable antibiotic-resistant marker gene *hygromycin phosphotransferase* (*hptII*) as plant selection markers. Rice callus induction, proliferation, *Agrobacterium-*mediated transformation, selection and regeneration were carried out as described by Hiei et al. [40] with some modifications [23]. The scutellum derived white, nodular, compact embryogenic calli were used for the transformation. The putative transformants were selected on selection medium containing 50 mg/l hygromycin B (Roche Diagnostics GmbH). Resistant calli were regenerated to proliferated green shoots. After differentiation and acclimatization, the putative transformed plants were grown in the greenhouse.

### PCR screening of putative tranformants

PCR amplification was used for the preliminary screening of transgenic plants. Genomic DNA was extracted from young green leaves of putative transformed and untransformed control rice plants following the CTAB extraction method [41]. PCR analysis was carried out using the gene specific primers (F1 and R1). For PCR analysis 100–200 ng genomic DNA was used as template and the reaction conditions were 94 °C for 5 min, 30 cycles of 94 °C for 30 s, 58 °C for 60 s and 72 °C for 60 s, with a final 7 min extension at 72 °C in My Cycler (Bio Rad, Hercules, CA, USA). The plasmid of binary vector construct, used for plant transformation (pCAMBIA130135S*mASAL*) served as the positive control while DNA from untransformed rice plants served as negative control. The amplification products were checked in 0.8 % agarose gel.

### Southern blot analysis

Southern blot hybridization was performed according to Sambrook et al. [42]. The genomic DNA (20 μg) from non-transformed and transformed plants was digested with restriction enzyme *Hin*dIII and electrophoresed on a 0.8 % (w/v) agarose gel and blotted onto positively charged nylon membrane (Hybond N+) (Amersham Biosciences) using the capillary transfer method, following depurination, alkali denaturation and neutralization. *mASAL* gene probe was prepared separately using “Ready Prime” random labelling system (Amersham Biosciences, UK) according to the manufacturer’s protocol. Then the membrane was hybridized overnight at 68 °C using [α^32^P] dCTP labelled *mASAL* gene probe (*Bam*HI and *Sac*I digested 333 bp *mASAL* fragment from pCAMBIA35S*mASAL*). After overnight hybridization the membranes were washed with 2X SSC (Merck, Germany), 0.1 % SDS (Sigma, USA) at room temperature for 45 min and at 68 °C for another 45 min using 0.1 % SSC, 0.1 % SDS. The membranes were then exposed to Kodak X-ray film for seven days at -80 °C and finally the films were developed.

### Segregation analysis of the transgene

T_1_ seeds collected from the self-pollinated T_0_ plants were germinated and DNA was isolated from one-month-old plants. PCR analyses for *mASAL* gene were carried out with gene-specific primers. The reaction mixtures were analyzed in 1.4 % agarose gel. After separation of the amplified product of the mASAL sequence, segregation patterns of the *mASAL* gene in progeny plants were calculated and validated by *χ*2 test.

### Western blot analysis

Total soluble protein was extracted from the fresh leaves of one-month-old untransformed and transformed rice plants in extraction buffer containing 20 mM Tris–HCl (pH 7.5) and 0.2 mM PMSF (phenylmethane sulfonyl fluoride) (Sigma, USA). The amount of protein in each sample was quantified by Bradford assay [43]. The total soluble protein (15 μg) from the individual line was separated on 15 % SDS-PAGE and electroblotted to positively charged Hybond C membrane (Amersham Biosciences). After blocking, the membrane was probed with anti-mASAL polyclonal primary antibody at 1:10,000 dilution followed by anti-rabbit IgG-horse radish peroxidase (HRP) conjugate (Sigma, USA) as secondary antibody at 1:20,000 dilutions. Bands were detected by enhanced chemiluminescence (ECL) reagents (GE Healthcare, Germany).

### ELISA of soluble protein extracts

The expression level of mASAL was quantified by ELISA. Wells of microtiter plates (Immunomaxi, Switzerland) were coated with 50 μg of total soluble protein extracted from transgenic leaves or purified mASAL serially diluted from 5 μg to 500 ng overnight at 4 °C in coating buffer (15 mM sodium carbonate, 35 mM sodium bicarbonate, 3 mM sodium azide; pH 9.6). The wells were blocked and then incubated with anti-mASAL primary antibody at 1:10,000 dilutions, followed by incubation with HRP conjugated anti-rabbit secondary antibody at 1:10,000 dilutions (Sigma, USA). Colour reaction was developed after addition of substrate *O*-phenylenediaminehydrochloride (Sigma, USA) dissolved in citrate buffer and the OD was recorded at 415 nm in a microtiter plate reader (ELx 800, Bio-Tek Instruments Inc., Winooski, VT, USA). All the blocking and washing steps were carried out according to Dutta et al. [22].

### Immunhistoflourescence analysis

Immunohistoflourescent localization of mASAL in transgenic plant tissue sections was performed according to the reported method of Yin et al. [44]. Hand sections of stems, leaves and roots from transformed as well as control plants were incubated in 10 % (v/v) trichloroacetic acid (Sigma, USA) at 4 °C for 1 h followed by ethanol:acetic acid (3:1, v/v) wash with three to four changes for complete removal of chlorophyll from green tissues. The tissue sections were then passed successively through series of graded ethanol to water (90 %, 70 %, 50 %, 30 % (v/v), respectively, each of 15 min duration) and blocked with 3 % (w/v) bovine serum albumin (Merck) in 1x phosphate buffered saline (PBS) at room temperature for 2 h. The tissue samples were incubated with an anti-mASAL antibody (1:10,000) in blocking solution overnight at room temperature. Finally, the sections were washed in 1x PBS followed by incubation with an anti-rabbit IgG-FITC conjugated (1:20,000) (Sigma, USA) secondary antibody for 1 h at room temperature. The slides were examined using an Axioscope Carl Zeiss inverted fluorescent microscope using excitation filter of 450–490 nm for FITC. Images were captured with the AxioCam ICc3 digital camera and the AxioVision imaging software system (Carl Zeiss Micro Imaging, GmbH, Germany).

### Bioassay using detached leaves

The non-transgenic controls, as well as the transgenic plants, were infected with *R. solani* culture after forty-five days post-transplantation to the soil in the greenhouse [45]. Bioassay using detached leaves was performed according to Kumar et al. [46]. Sterilized Petri plates were lined with thick sterile moistened cotton pads. The cotton was moistened periodically with sterile distilled water, to maintain the humidity. Sterile glass slides, with their ends inserted into slits cut 6 cm apart on a supporting Whatman 3 MM filter paper, were placed inside Petri plates. The fresh young leaves from both control and transgenic plants were assayed in this method. The leaf pieces were surface-sterilised with cut-ends inserted into the slits of the filter paper, keeping the abaxial surface up. Fungal mycelial disc (5 mm) scooped out from the peripheral region of 3-day-old PDA culture of *R. solani* was placed upon the middle of the leaf surface. The Petri plates were sealed with parafilm and kept at room temperature for 72 h. Moreover, the number of infection cushions on leaves of transgenic and non-transgenic plants was recorded after 72 hai. For studying the number of infection cushions both leaves of transgenic and non-transgenic control plants were stained with Trypan blue and Lactophenol (Himedia, India) and visualized using Axio Scope inverted fluorescence microscope (Carl Zeiss) under bright field.

### Whole plant bioassay

Sheath blight inoculation was performed according to the method described previously [37]. *R. solani* (maintained on PDA at 28 °C) was inoculated into potato dextrose broth (PDB) and incubated on a 28 °C shaker for 72 h. Mycelia were collected and separated into 5-mm-diameter balls. Each mycelial ball was secured against the sheath of rice plants by aluminium foil. Sterile water was sprayed regularly to maintain a humid environment. The development of symptoms caused by *R. solani* infection was recorded after 7, 14 and 21 days of inoculation and graded using a scale ranging from 0 to 9. The scale was based on the relative lesion height on the whole plant, according to the Standard Evaluation System for Rice [47]. Based on the Standard Evaluation System, disease intensity was expressed as the PDI on transgenic and control wild-type plants [35].

### Statistical analysis

The data were analysed using Graphpad prism 5 software (GraphPad Software, La Jolla, CA, USA). One-way analyses of variance (ANOVA) were used to compare the differences between the non-transgenic control and the transgenic plants. *P* < 0.05 was considered to be statistically significant.

## Abbreviations

ANOVA: analysis of variance
dpi: days post-inoculation
ELISA: enzyme-linked immunosorbent assay
hai: hours after inoculation
*hptII*: hygromycin phosphotransferase
mASAL: mutant *Allium sativum* leaf agglutinin
PBS: phosphate buffered saline
PDA: potato dextrose agar
PDB: potato dextrose broth
PDI: percentage disease index
